# Mechanisims of asthma and allergic disease – 1079. Evidence of platelet activation in asthmatic patients

**DOI:** 10.1186/1939-4551-6-S1-P75

**Published:** 2013-04-23

**Authors:** Hyun Jung Jin, Nami Shrestha Palikhe, Ji Won Lee, Mi-Ae Kim, Yoo Seob Shin, Young Min Ye, Hae-Sim Park

**Affiliations:** 1 Department of Internal Medicine, College of Medicine, Yeungnam University, Daegu, South Korea; 2 Pulmonary Research Group, Department of Medicine, University of Alberta, Canada; 3 St. Marks School, Southborough, USA; 4 Department of Allergy & Clinical Immunology, Ajou University School of Medicine, Suwon, South Korea; 5 Allergy and Clinical Immunology, Ajou University Hospital, South Korea

## Background

Animal models of allergic asthma indicate that intravascular platelet activation is essentail for the development of allergen induced chronic airway inflammation. P2Y12, the third CysLT receptor, is expressed on platelets and has been an important pathophysiological role in LTE4 mediated pulmonary inflammation. We investigated platelet activation status in asthmatic patients compared to controls.

## Methods

Fifty asthmatic patients and 20 healthy controls were enrolled from Ajou University Hospital, Suwon, Korea Surface expression of P-selectin and P2Y12 on platelets were determined by flowc ytometry. Plasma soluble P-selectin level was measured by ELISA. The asthmatic subjects were classified into two groups depending on high (>mean + 2 SD of controls) and low expression of P2Y12.

## Results

The expressions of platelet P-selectin, P2Y12 and soluble p-selectin level were significantly higher in asthmatic patients than in controls, (*p*<*0.001*, *p*=*0.001*, *p*<*0.001*, respectively). No significant correlations were found between clinical parameters and platelet activation markers. Expressions of P2Y12 on platelet did not increase significantly after the treatment with LTE4 or aspirin. Higher expression group of P2Y12 had significantly higher peripheral eosinophil count (*P*=0.021).

## Conclusions

Platelet activation may play a role in asthma pathogenesis. A possible interaction between platelet and eosinophil via P2Y12 was suggested.

